# Comment to the Letter to the Editor entitled “Lithium in drinking water and suicide risk” by Tomoyuki Kawada

**DOI:** 10.17179/excli2022-5227

**Published:** 2022-08-03

**Authors:** Antonina Luca, Maria Luca

**Affiliations:** 1 Department Medical, Surgical Sciences and Advanced Technologies, "GF Ingrassia", University of Catania, Italy; 2 Center for Addiction, via Pò n.2, Adrano, Catania, Italy

## ⁯⁯⁯

Lithium is a metal naturally present in the Earth's crust. It is contained in traces in all soils and in higher amount in grains and vegetables. Since the mid-20^th^ century, due to its proven efficacy in a plethora of psychiatric disturbances, including bipolar disorder (Fountoulakis et al., 2022[4]), recurrent unipolar depression (Undurraga et al., 2019[9]), suicide (Del Matto et al., 2020[2]; Girlanda et al., 2014[5]) and personality disorders (Luca et al., 2012[7]), lithium carbonate still represents one of the most prescribed drugs in psychiatry. 

Interestingly, although the oral therapeutic doses of lithium ranges from 600 to 1200 mg per day, previous studies suggested that the beneficial effect of lithium as mood stabilizer may be exerted at significantly lower doses, such as those naturally found in the environment (Young, 2009[10]). Actually, several ecological studies have assessed the association between the presence of lithium in drinking water and suicide risk in the general population, generally reporting an inverse association, particularly in men (Barjasteh-Askari et al., 2020[1]). 

In the Letter to the Editor entitled “Lithium in drinking water and suicide risk”, Tomoyuki Kawada (2022[6]) called for caution when interpreting ecological data, because associations recorded at a population-based level may not exert the same effect on an individual-based level. We strongly agree with Kawada's considerations. Moreover, although the inverse association between lithium concentration and suicide risk has been more frequently confirmed in men, the aforementioned studies did not perform a sex-stratified analysis to better describe the phenomenon. The explanation of the reported sex-related difference in lithium associated lower risk of suicide is far to be easy to delineate. Lithium bioavailability is in fact influenced by gastric secretions, weight, body fat percentage, hepatic metabolism and renal elimination and sex hormones (Flores-Ramos et al., 2017[3]). Furthermore, the higher incidence of suicide among men, regardless lithium intake, should be considered when interpreting these data. Finally, considering that suicide represents the result of different genetic, biological, demographic, psychiatric and environmental factors (McMahon et al., 2022[8]), the role of potentially confounder variables (i.e. economic availability, marital status, personality disorders, previous suicide attempts, previous psychiatric diagnosis…) in the association between water lithium intake and suicide risk should not be underestimated. In light of these aspects, risk-benefit considerations must be made when hypothesizing the supplementation of tap water with low concentration of lithium. Indeed, despite this procedure would represent a cheap and potentially beneficial opportunity to reduce suicide risk, lithium safety for pregnant women, people already assuming lithium carbonate and people suffering from thyroid or kidney disorders must be carefully considered. 

## Declaration

### Conflict of interest

The authors declare that they have no conflict of interest.

### Financial disclosures

None.
